# Psychosocial Aspects of Infertility and Medically Assisted Reproduction in Serbia: A COMPI-Based Single Centre Study

**DOI:** 10.3390/healthcare14101429

**Published:** 2026-05-21

**Authors:** Lidija Tulic, Jelena Dotlic, Tatjana Madic, Dejan Uljarevic, Aleksandar Dmitrovic, Lone Schmidt, Mariana Veloso Martins, Jelena Stojnic, Jelena Micic, Jovan Bila, Dragisa Sljivancanin

**Affiliations:** 1 Clinic of Obstetrics and Gynecology, University Clinical Center of Serbia, Dr Koste Todorovica 26, 11000 Belgrade, Serbia; 2 Medical Faculty, University of Belgrade, Dr Subotica 8, 11000 Belgrade, Serbia; 3 Section of Social Medicine, Department of Public Health, University of Copenhagen, 1165 Copenhagen, Denmark; 4 Faculty of Psychology and Education Sciences, University of Porto, 4200-135 Porto, Portugal

**Keywords:** infertility, COMPI scale, stress, coping strategies, Serbia

## Abstract

**Background:** Infertility affects millions of people causing grave societal and health consequences (poor physical and mental wellbeing). **Aims:** To translate and validate the COMPI scale in Serbian and examine associations of infertility-related stress, coping strategies and evaluation of care with medically assisted reproduction (MAR) outcomes in female Serbian infertility patients. **Methods:** The study included patients undergoing MAR for four months. Participants completed a socio-demographic and gynecologic questionnaire, the Serbian-translated COMPI scale, Beck Anxiety Inventory (BAI) and Zung Self-Rating Depression scale (ZDS). Serbian COMPI was validated in the classic manner. Associations between COMPI scores and pregnancy outcomes were analyzed by Spearman’s correlation and multivariable regression. **Results:** A total of 107 women participated and 24.3% achieved pregnancy. The Serbian COMPI demonstrated high internal consistency (Cronbach alpha = 0.838). Compared with reference COMPI data, personal, social and marital stress scores were higher, while meaning-based coping and marital benefit scores were lower. Regression analysis showed that higher marital stress, partner communication difficulties and meaning-based coping were associated with higher pregnancy likelihood. **Conclusions:** Serbian patients undergoing MAR reported high infertility-related stress and predominantly used active coping strategies. Patients who applied meaning-based coping were more likely to achieve pregnancy.

## 1. Introduction

The problem of infertility worldwide affects millions of people, causing grave societal (social stigma, economic difficulties) and health consequences (poor physical and mental wellbeing). Numerous factors negatively affect reproduction. Conversely, advances in medical science and technology, such as medically assisted reproduction (MAR), have became a major therapeutic approach with a high success rate for achieving pregnancy [1,2].

Infertility is often addressed primarily as a medical problem, while its psychological aspects have often been overlooked. Current understanding however recognizes infertility as a multifactorial condition in which psychological and social factors play a significant role [3]. Receiving a diagnosis of infertility causes significant stress, with a substantial impact on quality of life. Infertility-related stress adversely affects partner relationships and social interactions [4,5]. In some communities, life without children can be especially difficult as infertility patients often experience not only personal grief but also social judgment, inadequate understanding of their condition and treatment needs, cultural pressure to conform to traditional parenthood norms, and potential marital strains resulting from these pressures. These factors can exacerbate psychological distress and contribute to social marginalization and even stigmatization, resulting in isolation and emotional distress [6,7].

Couples facing infertility often turn to MAR. However, this process can be highly stressful and burdensome due to prolonged therapy and medical interventions, as well as the anxiety of waiting for results. Therefore, the MAR process may heighten stress and contribute to considerable emotional and psychological difficulties for both partners [8,9]. It has been suggested that stress, anxiety and depression may reduce the likelihood of a successful MAR outcome, while other investigations did not prove that stress can cause adverse outcomes of pregnancies after MAR. Therefore, the mechanisms and extent to which stress influences MAR success are yet to be clarified [10,11].

Given the recognized impact of psychosocial well-being on both quality of life and treatment outcomes, standardized assessment tools are needed. The Copenhagen Multicentre Psychosocial Infertility (COMPI) Research Program developed an instrument to evaluate infertility-related stress, coping strategies, communication patterns and patient attitudes toward fertility treatment [12]. The COMPI scale was developed in English and, to date, has been translated and culturally adapted for several populations. However, no translation exists for the Serbian language [13].

Despite the growing body of mental health research within infertility and MAR worldwide, little is known about the psychological experience of infertility in Serbia. In Serbia, MAR has become increasingly accessible, but psychosocial care remains inconsistently integrated into clinical practice and is mostly sought by patients individually [14]. Given the limited number of studies examining the impact of infertility on stress and mental health in the Serbian population, there is a clear need to validate an instrument that could facilitate more effective treatment and support for Serbian infertility patients. Additionally, the availability of a validated scale would enable a greater number of investigations, thus providing more accurate data and new scientific insights into infertility-related stress and coping strategies among Serbian patients. Finally, this data could be compared with other populations worldwide in a standardized manner.

The aim of this study was to translate and validate the COMPI scale in Serbian, identify and assess key psychosocial variables in women undergoing MAR procedures, and examine associations between these psychosocial factors and treatment outcomes.

## 2. Materials and Methods

All consecutive women and their partners that were enrolled in MAR procedures (intrauterine insemination—IUI, in vitro fertilization—IVF and intracytoplasmatic sperm injection—ICSI) at the Clinic for Ob/Gyn University Clinical Center of Serbia between September and December 2024 were invited to participate in this study. However, due to the low response rate of male partners (26.2%), this paper presents results of female infertility patients only. Before commencing, the MAR patients were asked to complete the questionnaires chosen for the study. All patients were thoroughly informed about the research and all MAR procedures after which they signed the informed consents for inclusion in the study as well as the MAR itself. The study was conducted in accordance with the Declaration of Helsinki, and approved by the Ethical Committee of the University Clinical Center of Serbia (No 825/1-P).

The inclusion criteria for the study corresponded with the criteria for MAR: being 18 to 40 years old and independent (not having a legal guardian); having a Body Mass Index (BMI) of 18 to 30 kg/m^2^, an established diagnosis of infertility (tubar, ovarian, male or unknown factor) and agreeing to participate in the study. Infertility was diagnosed and classified according to European Society of Human Reproduction and Embryology (ESHRE) guidelines [15]. All MAR procedures were carried out in accordance with relevant guidelines and regulations. Achieving clinical pregnancy (ultrasound visualization of viable intrauterine pregnancy in the 6th gestational week) was considered as successful MAR outcome.

### 2.1. Instruments

Participants completed a socio-demographic and gynecologic questionnaire (age, level of education, employment, marital status, reproductive history, previous infertility diagnostics and treatments).

The COMPI scale is a specific instrument for assessing different infertility-related issues in couples seeking treatment [12]. It was designed upon a prospective longitudinal cohort study on a large population of infertile couples with an aim to evaluate the fertility treatment process and its psychosocial consequences. The COMPI scale has demonstrated good reliability and cultural applicability in several countries but has not yet been validated or applied in a Serbian MAR population [16,17,18,19]. The permission to translate, validate and use the scale in our research was obtained from the authors.

The COMPI scale was translated according to the internationally accepted methodology. Translation from the original English to Serbian language (“forward translation”) was performed by two independent translators (study authors) who are native Serbian speakers and fluent in the English language. The “backward translation” (from Serbian back to English) was completed by the third translator (native English speaker fluent in Serbian language) who was blinded to the original questionnaire. Finally, the language coordinator, together with a clinical expert from the team, discussed all translations and created the final version of the Serbian COMPI scale which was likely to be the most appropriate for the cultural environment of Serbia.

The scale has 71 items divided into 7 subscales. The responses are marked on a Likert scale. For each subscale, the total score is calculated as the mean of item grades. Higher scores indicate more stress [12].

The Fertility Problem Stress subscale measures the amount of stress the fertility problem places on everyday life. It has 14 items grouped into 3 domains: personal stress domain (6 items; PD score range 6–26; infertility-related stress impact on mental and physical health), marital stress domain (4 items; MD score range 4–18, infertility impact on the marital and sexual relationships) and social stress domain (4 items; SD score range 4–16, influence of infertility-related stress on social relationships) [12].

The Coping Strategy subscale evaluates the methods for managing infertility-related issues. It has 19 items categorized into four domains: active-avoidance coping (4 items; AACOP score range 4–16; avoiding pregnant women or children); active-confronting strategies (7 items; ACCOP score range 7–28; showing feelings, asking for advice); passive-avoidance strategies (3 items; PACOP score range 3–12; disregarding infertility, hoping for a miracle); and meaning-based coping (5 items; MBCOP score range 5–20; positive personal development, finding other goals in life) [12].

The Infertility-related Communication Strategies subscale (ICS scale score 6–18) assesses whether partners talk about their infertility problems with others (formally or open-mindedly) or are they secretive about it. The Partner Communication subscale (PC) examines the willingness of the partner to discuss infertility issues. The Marital Benefit Measure subscale (MBEN) investigates whether not having children has strengthened or caused problems in the relationship of investigated couples [12].

Patients’ attitudes to and evaluation of fertility treatment are two separate subscales that investigate what medical and psychosocial services fertility patients expect from the medical system and how they evaluate fertility treatment. Patients are first asked about the perceived importance of medical care (4 items; MCAT score range 4–12), patient-centered care (4 items; PCAT score range 4–12) and professional psychosocial services (8 items; PPAT scale sore 8–28) and then they evaluate medical care (7 items; MCEV score range 7–42) and patient-centered care (6 items; PCEV score range 6–36) they received [12].

The Beck Anxiety Inventory (BAI) is a self-reported scale designed to measure the magnitude of clinical anxiety. It provides quantitative information about how people react to possible sources of maladaptive emotional reactions and minimizes the overlap between anxiety and depression. The BAI consists of 21 anxiety symptoms which are graded on a Likert scale (0—not bothered to 3—severely bothered by the symptom). Higher total scores (the sum of item grades) indicate more severe anxiety symptoms. The standardized cutoffs are 0–7 minimal; 8–15 mild; 16–25 moderate and 26–63 severe anxiety [20].

The Zung Self-Rating Depression Scale (ZDS) is a short self-administered survey for assessing and quantifying the depressed status of a patient. The scale has 20 items that present the four typical characteristics of depression. Items are scored on a Likert scale (1—little of the time to 4—most of the time) to describe how often patients felt or behaved in the mentioned way. Higher total scores (the sum of item grades) indicate more severe depressive symptoms. The standardized cutoffs are ≤49 normal range; 50–59 mildly depressed; 60–69 moderately depressed; ≥70 severely depressed [21].

### 2.2. Patient and Public Involvement

Patients were involved in the research through study design or conduct as well as the analysis and interpretation of their data.

### 2.3. Statistical Analysis

The data were analyzed by means of descriptive (range, mean, standard deviation, frequency, percent) and analytical statistics using SPSS 20.0 for Windows (IBM, Armonk, NY, USA) and JASP 19.3.

Initially, the validity and reliability of the Serbian version of COMPI scale were tested. Internal consistency of the Serbian COMPI was evaluated using Cronbach alpha coefficient (satisfactory value is above 0.7). Discriminating characteristics of the scale items were tested by Corrected Item—Total Correlation (CI–TC) analysis. An item is considered as an adequate part of the questionnaire if CI–TC is ≥0.40 [22]. In exploratory factor analysis (EFA), the minimum residuals factoring method was applied to extract the factors. Additionally, parallel analysis was performed to ensure that the selection of latent factors is properly conducted. In the confirmatory factor analysis (CFA), as the model goodness-of-fit estimators, we assessed goodness of fit index (GFI), comparative fit index (CFI), root mean square error of approximation (RMSEA) and SRMR (Standardized Root Mean Square Residual). For the model to be adequate values of RMSEA and SRMR, indices should be below 0.1 while other indices should be optimally higher than 0.90 [22]. The concurrent validity was investigated by assessing correlations between COMPI subscale and domain scores and Beck Anxiety Inventory and Zung Self-Rating Depression scale scores [22].

To compare patient characteristics and investigated scale scores regarding pregnancy achievement, the Kruskal–Wallis *χ*^2^ test and ANOVA were applied. Spearman’s correlation was utilized to investigate the associations of COMPI subscale and domain scores between each other and with patient characteristics. Finally, regression analysis was performed to assess the associations of COMPI subscale and domain scores with pregnancy achievement. The model was adjusted for BAI and ZDS scores [22].

## 3. Results

The study included 107 female infertility patients undergoing MAR (response rate 87.7%) who on average were 35.06 ± 3.91 years of age. The MAR success rate according to the current literature and patients’ age range (18–40) should be 37.5%. Consequently, the study power was 83.4% (alpha error 0.05).

The majority of the investigated women were highly educated and employed. The unknown cause of infertility was the most common (31.8%) while another 31.8% of patient couples had issues with male infertility. The examined women mostly had primary infertility lasting for 5.21 ± 2.93 years and up to four previous MAR procedures. Still, only 11 women already had children. In the examined MAR cycle, clinical pregnancy was achieved in 26 (24.3%) patients, while significantly more patients (81; 75.5%) had unsuccessful MAR (*p* = 0.001). Patient and MAR data are presented in Table 1 and Table 2.

Significantly more patients who already had a successful pregnancy managed to achieve clinical pregnancy now as well. Still, the majority of patients did not have successful MAR outcomes, both previously and currently (*p* = 0.005). No other significant differences between patients who had and had not achieved clinical pregnancy in the current MAR cycle were registered.

The Serbian version of the COMPI scale was proven as a valid and reliable instrument (Cronbach coefficient alpha 0.838; McDonald omega 0.766, CI–TC ≥ 0.40 for all items). Exploratory factor analysis showed that the COMPI seven-factor structure in the Serbian population explained 54.84% of the variance. Parallel analysis confirmed that seven-factor structure should be retained in the Serbian version of COMPI as well as in the original (Table 3). Confirmatory factor analysis proved an adequate construct of Serbian COMPI (CFI 0.913, GFI 0.966, SRMR 0.970 and RMSEA 0.1).

In our study, COMPI subscale and domain scores had wide ranges. According to the referral ranges of scores from the literature, all subscale and domain scores obtained in our study had both the lowest and the highest values.

As for the mean values of domain scores, AACOP, SD and MD had significantly, while ACCOP and PD had slightly higher means than those from the literature (Table 2). Therefore, it can be seen that our patients had significant levels of infertility-related stress. Coping by active avoidance and by active confronting were the usual strategies for dealing with infertility issues and stress among our patients. Contrarily, the obtained mean values of PACOP, MBCOP and MBEN were lower than those from the literature (Table 2). This indicated that among our patients, passive and meaning-based coping were less common stress coping mechanisms. The marital benefits of infertility treatment for our patients were not as good as in other populations.

Both the highest and mean scores of almost all domains regarding treatment attitudes and evaluation were significantly lower in our sample than those from the literature (Table 2). The only aspect of the therapeutic approach that was highly scored by our patients indicating that it was perceived as important was professional psychosocial service.

Significant differences between patients who achieved and did not achieve clinical pregnancy were registered only for MD, MBCOP and PC scores (Table 2). Patients who achieved clinical pregnancy more often used meaning-based coping strategies to overcome infertility-related stress. Investigated patients tried to be positive about infertility and its treatment in almost 80%. Still, these patients also reported having more marital stress and more difficulties in communication with partners. In our overall sample, more than 70% of patients reported that infertility and childlessness caused some marital problems while only around 5% of couples have strengthened their relationship.

Supplementary Tables S1 and S2 present the correlations of COMPI scale domain scores between each other, with BAI and ZDS as well as with the characteristics of the examined patients. Few associations of scale scores with investigated socio-demographic and medical history characteristics of our patients were registered, but significant correlations of COMPI subscale and domain scores with BAI and ZDS were present.

A higher active-avoidance coping strategy score correlated with higher ZDS. A higher active-confronting coping strategy score correlated with not having any previous deliveries, but having previous miscarriages and not receiving any therapy for infertility before. A higher passive-avoidance coping strategy score correlated with a higher level of education and not receiving any therapy for infertility before. A higher meaning-based coping strategy score correlated with achieving pregnancy, higher scores of BAI and ZDS, having previous miscarriages and not receiving any therapy for infertility before.

A higher marital stress domain score correlated with achieving pregnancy now and before, not being married and surgical therapy for infertility cause. A higher social stress domain score correlated with higher scores of BAI and ZDS, not achieving pregnancy after infertility treatment before and surgical therapy for infertility cause.

More difficulties in communications with partners correlated with achieving pregnancy, higher ZDS and being in a committed relationship or marriage. Better infertility-related communication correlated only with an older age of patients. A higher marital benefit domain score correlated with higher scores of BAI and ZDS, having previous pregnancies and miscarriages.

Patients having ICSI compared to those having surgical treatment for infertility and/or intrauterine inseminations perceived patient-centered care as more important. Patients with lower ZDS and those who had ICSI considered their treatment to be of high-quality.

Finally, a significant regression model was obtained when we assessed the associations of COMPI scale scores and clinical pregnancy achievement. It showed that patients who reported more marital stress and more difficulties in patient communications but applied meaning-based coping strategies to deal with infertility issues and stress were more likely to achieve clinical pregnancy (Table 4).

## 4. Discussion

Studies on the potential influence of psychological factors on MAR outcomes have shown substantial heterogeneity that may obscure potential associations between stress and MAR outcomes [23]. This discrepancy can be attributed to differences in population characteristics, study design and assessment methods for psychosocial variables. Prior research varies widely in socio-demographic characteristics such as the mean age of women, duration of infertility, infertility etiology and timing of stress assessment—whether at treatment entry or during treatment [10,24].

In our sample, infertility-related stress was particularly high in the personal, social and marital domains, with active avoidance and active confronting as the most common coping strategies. Our COMPI data revealed that investigated patients reported higher scores for active-avoidance and active-confronting coping strategies compared to reference values from other populations, while passive and meaning-based coping were less frequently used [12].

Infertile couples often experience severe stress related to diagnosing and treatment of their condition which leads to numerous changes in their personal lives, couple and sexual relationships, social networks, financial resources and life expectations. Infertility can profoundly impact emotional well-being, negatively affect self-perception and view of life, often causing a perceived loss of control over one’s life. It can dominate daily thoughts and conversations, leading to frustration, diminished self-esteem and feelings of inadequacy and repeated failure [25,26,27]. Therefore, they need to implement different coping strategies as means of psychological adjustment. Coping is defined as conscious and voluntary activation of thoughts, emotions and behaviors used to manage internal and external stressful situations and it can be reactive (a reaction after the stressor) and proactive (aiming to neutralize future stressors). In some studies, the most common coping strategies included religious and spiritual methods, emotional (expressing feelings) and problem-focused (learning about treatment) coping and seeking social support, although passive avoidance was also frequent [25,26,27].

Higher social stress scores in our study were associated with undergoing surgical therapy for infertility, failure to achieve pregnancy after previous infertility treatments and elevated anxiety and depression. Inability to achieve pregnancy even after MAR attempts is likely to increase social pressure as well as the feelings of isolation and stigmatization [4,6]. Psychological distress is often exacerbated by perceived or actual social pressure and rejection. Partner and family support are vital, but lack of empathy from family and friends, discrimination or even violence against infertile women remain a major issue in some cultural settings [12]. One of the most difficult social aspects reported by infertile women is interacting in environments where pregnant women and children are present, often triggering jealousy or sadness [25,26].

Marital benefit scores for our patients were notably lower than in prior studies. This may reflect the influence of cultural norms, social stigma and traditional gender roles in Serbia, as well as the significant marital stress reported by over 70% of participants. In this context, infertility is often perceived as both personal and family failure, which can place a disproportionate emotional burden on women and strain marital relationships [8,25,26]. Additionally, societal expectations regarding parenthood may reduce the perceived supportive aspects of marriage when reproductive goals are not met. Secrecy about infertility, difficult marital communication and active-avoidance coping were linked to lower marital benefit at 12 months. Still, those patients that had higher marital benefit scores had greater anxiety and depression, but also more previous pregnancies and miscarriages. Moreover, patients with higher marital stress scores were more likely to have previous pregnancies as well as to achieve pregnancy in the examined MAR cycle [28,29]. Moreover, difficulties in communication with a partner were also associated with achieving pregnancy, as well as with higher depressive symptomatology and being in a committed relationship or marriage. Although these findings are somewhat unexpected, they may reflect the dual nature of infertility as both a source of distress and an opportunity for couples to deepen their bonds through shared struggle. While high marital stress is usually seen as detrimental, couples more emotionally engaged in treatment may report high marital stress and frequent arguments [29,30]. Couples engaged in active treatment may experience strain in communication due to the emotional intensity of the process, yet still work collaboratively toward the shared goal of conception. In this “engaged under strain” profile, relational friction coexists with intensive involvement in care, adherence to demanding treatment schedules and willingness to pursue advanced interventions (e.g., surgical therapy), all of which may contribute to higher success rates [25,26,30]. On the other hand, in our study, better infertility-related communication was associated only with older patient age, which may indicate greater relationship maturity and experience in navigating emotionally charged topics.

The literature evidence connecting coping to pregnancy outcomes is mixed and may reflect nuanced, treatment-engaged adaptation rather than a simple “stress causes failure” narrative [11,28]. Some studies suggested that general emotional distress is not a consistent determinant of MAR success, while others have reported associations between specific coping styles and outcomes (emotionally expressive coping linked to lower and meaning-based coping linked to higher pregnancy rates) [27,31]. Our study results showed that patients who achieved pregnancy in the current cycle more often used meaning-based coping, but paradoxically also reported higher marital stress and greater difficulties in partner communication. This pattern may suggest that patients who are able to reframe infertility in a positive light can still experience relationship strain, but perhaps channel all issues into adaptive motivation for treatment adherence and emotional resilience [25,26,32]. Moreover, some authors also found that stress does not directly impact the biological processes of implantation and pregnancy rates after MAR [33]. Such findings correspond with the literature linking coping styles to stress trajectories and relationship quality, with meaning-based coping often associated with lower stress over time and active avoidance to higher stress and reduced marital benefit. A rather unexpected finding that higher marital stress and greater difficulties in partner communication are associated with pregnancy achievement could lie in fact that only women were included in this study. The cultural settings in Serbia often make the female partner the one (occasionally the only one) responsible for the whole process of MAR. This can lead to general and marital stress, but also can lead to more commitment by the female to collaborate with her gynecologists in order to achieve the desired success. However, this explanation is highly speculative and should be confirmed optimally through further qualitative studies. Meaning-based coping, by enabling reappraisal and emotional reframing, may help sustain engagement despite interpersonal strain. Finding meaning in the experience generally reduces stress, while avoidance increases it [34,35].

Women frequently report infertility evaluation and treatment as one of the most distressing life events causing a high prevalence of depressive symptoms [36,37]. Our patients strongly endorsed psychosocial services but evaluated received care rather low. Notably, lower depressive symptoms and undergoing ICSI were both associated with higher perceived treatment quality, which may indicate that both psychological well-being and access to advanced procedures shape patients’ treatment perceptions. Such findings were somewhat different from previous data in which satisfaction with fertility treatment was commonly high in different populations [31,38]. The study results suggest that counselling is not consistently and adequately integrated into MAR programs in Serbia.

The obtained results emphasize the complex interplay between stress, coping strategies and MAR outcomes. While certain stressors may be detrimental, others when coupled with adaptive coping strategies such as meaning-based reframing may enhance persistence and treatment adherence, ultimately improving chances of pregnancy. This highlights the need for integrating structured psychosocial support into infertility care, both to reduce distress and strengthen adaptive coping mechanisms to improve both emotional well-being and reproductive outcomes [32,39].

The study strengths include use of standardized validated psychosocial instruments and rigorous psychometric evaluation of the Serbian COMPI. Use of multiple COMPI subscales alongside anxiety and depression measures enabled multidimensional assessment of infertility experience. Adjustment for relevant covariates in regression models added robustness.

Clinical implications include the potential value of routine psychosocial screening by COMPI scale at MAR entry, identification of patients with high marital or social stress for targeted support and integration of meaning-based coping interventions into MAR programs. As investigated patients rated professional psychosocial services as highly important but evaluated them less positively, employing trained mental health professionals in fertility clinics could help bridge this gap.

One of the study limitations was cross-sectional design. Therefore, only associations could be studied and not their direction and effect. The study was conducted at a single center over a relatively short period, which may have reduced statistical power for detecting smaller effect sizes and limited generalizability.

Moreover, the study design caused the rather low sample size. Larger samples (around 300 participants) are generally recommended for psychometric validation. The number of participants for adequate factor analysis should be at least five times larger than the number of items in the instrument (in our case 355 participants for 71 COMPI items). However, for our initial single center study, we opted to have a sample that reflects consecutive recruitment of a well-defined clinical population.

Finally, although male partners were invited, they were less willing to participate and therefore their opinions were not included in analysis, which also lowered the overall sample size.

The low sample size could have influenced the obtained results such as Cronbach’s alpha causing the reliability to be moderate. Consequently, the representativeness of the obtained results is limited and their generalizability should be made with caution. Additionally, the findings of factor analysis should not be considered as absolutely valid as they were performed on a small sample. Consequently, they should not be generalized before confirmation in further larger investigations.

Another study limitation caused by the small sample is the potential overfitting of the regression model. Still, we opted to test all 15 domains and subscale COMPI scores. Moreover, the regression model was adjusted for BAI and ZDS scores, leading to a total of 17 potential predictors. For reliable regression results, the sample should have included at least 170 patients. Consequently, the obtained model and determined predictors should be carefully interpreted and applied in practice only after confirmation on larger samples.

Furthermore, it should be noticed that as the study outcome we assessed a single-cycle MAR success. This might have impacted the results as it is well known that to achieve pregnancy in MAR often requires multiple cycles. Additionally, the COMPI scale is based on self-reporting which can cause possible recall bias. Finally, at this point we did not statistically compare our results with the literature data. The presented comparisons are made solely as the part of the discussion and interpretation of the obtained scores.

Future research should be performed on larger multicenter samples as well as in samples of women with different socio-epidemiological backgrounds and causes of infertility. This research should also employ longitudinal stress, coping and relationship quality assessment before, during and after MAR. Including both partners will provide a fuller understanding of dyadic coping and its impact on outcomes. Finally, the obtained results for Serbian population could be compared with other populations worldwide.

## 5. Conclusions

In conclusion, the investigated Serbian female fertility patients reported high levels of infertility-related stress, while they generally used active avoidance and active confronting as strategies for dealing with infertility issues and stress. Patients who reported more marital stress and more difficulties in patient communications but applied meaning-based coping strategies to deal with infertility issues and stress were more likely to achieve pregnancy. Our findings emphasize the need for integrated psychosocial services in Serbian MAR programs. The Serbian version of the COMPI scale was found to be a reliable instrument and therefore should be implemented in clinical practice for assessing infertility-related stress and treatment satisfaction.

## Figures and Tables

**Table 1:** Socio-demographic and medical history data of investigated infertility patients.

Parameters		Overall Sample		No Pregnancy		Achieved Pregnancy	
		Number	Percent	Number	Percent	Number	Percent
Education	Primary	5	4.7	4	4.9	1	3.8
	Secondary	37	34.6	25	30.9	12	46.2
	High	65	60.7	52	64.2	13	50.0
Employment	No	22	20.6	17	21.0	5	19.2
	Yes	85	79.4	64	79.0	21	80.8
Marriage	No	24	22.4	18	22.2	6	23.1
	Yes	83	77.6	63	77.8	20	76.9
Pregnancy before	No	72	67.3	58	71.6	14	53.8
	Yes	35	32.7	23	28.4	12	46.2
Previous pregnancy outcome	Miscarriage	17	48.6	12	14.8	2	16.7
	Ectopic	7	20.0	5	6.2	5	41.7
	Delivery	11	31.4	6	7.4	5	41.7
Infertility cause	Tubes	29	27.1	21	25.9	8	30.8
	Ovary	10	9.3	7	8.6	3	11.5
	Male	34	31.8	25	30.9	9	34.6
	Unknown	34	31.8	28	34.6	6	23.1
Therapy before	No	29	27.1	20	24.7	9	34.6
	Yes	78	72.9	61	75.3	17	65.4
Therapy type before	Female surgery	20	25.6	15	24.6	5	29.4
	Male surgery	9	11.5	7	11.5	2	11.8
	IUI	30	38.5	26	42.6	4	23.5
	IUI donation	1	1.3	1	1.6	0	0
	IVF	13	16.7	8	13.1	5	29.4
	ICSI	5	6.4	4	6.6	1	5.9
Pregnancy after therapy	No	64	82.1	54	88.5	10	58.8
	Yes	14	17.9	7	11.5	7	41.2
Infertility therapy now	Female surgery	14	13.1	10	12.3	4	15.4
	Male surgery	2	1.9	2	2.5	0	0
	IUI	10	9.3	8	9.9	2	7.7
	IUI donation	5	4.7	3	3.7	2	7.7
	IVF	70	65.4	52	64.2	18	69.2
	IVF donation	2	1.9	2	2.5	0	0
	ICSI	4	3.7	4	4.9	0	0
Parameters		Mean	Standard Deviation	Mean	Standard Deviation	Mean	Standard Deviation
Age		35.06	3.91	35.25	3.92	34.46	3.89
Infertility years		5.21	2.93	5.33	2.93	4.81	2.98
Therapy number		2.24	1.71	2.27	1.71	2.11	1.79

Legend: IUI—intrauterine insemination; IVF—in vitro fertilization; ICSI—intracytoplasmatic sperm injection.

**Table 2:** COMPI infertility, Beck Anxiety and Zung Depression scale scores.

Parameters and Scales	Overall Sample		No Pregnancy		Achieved Pregnancy		Between Groups *p* Per Scale Score
	Mean	Standard Deviation	Mean	Standard Deviation	Mean	Standard Deviation	
PD	12.63	2.70	12.74	2.84	12.31	2.21	0.479
MD	9.11	1.83	8.85	1.87	9.92	1.49	0.009
SD	5.90	2.87	6.19	3.01	5.01	2.19	0.064
AACOP	12.27	3.07	12.08	3.09	12.84	2.97	0.275
ACCOP	17.98	5.19	17.81	5.12	18.51	5.46	0.561
PACOP	6.56	2.48	6.56	2.47	6.53	2.54	0.958
MBCOP	10.56	3.90	9.86	3.56	12.73	4.18	0.001
PC	2.70	0.51	2.64	0.55	2.88	0.32	0.037
ICS	11.84	2.39	11.67	2.33	12.34	2.54	0.218
MBEN	3.88	2.21	3.96	2.38	3.65	1.54	0.538
MCAT	5.28	1.35	5.25	1.37	5.38	1.32	0.685
PCAT	6.33	1.77	6.29	1.71	6.46	1.98	0.682
PPAT	15.60	5.04	15.18	4.91	16.92	5.34	0.127
MCEV	10.48	2.51	10.41	2.47	10.69	2.67	0.633
PCEV	7.70	1.88	7.64	1.91	7.88	1.79	0.570
Beck Anxiety	7.85	7.97	7.91	8.61	7.73	5.66	0.925
Zung Depression	34.43	7.65	34.71	8.01	33.57	6.54	0.512

Legend: PD—personal stress domain; MD—marital stress domain; SD—social stress domain; AACOP—active-avoidance coping; ACCOP—active-confronting strategies; PACOP—passive-avoidance strategies; MBCOP—meaning-based coping; ICS—infertility-related communication strategies; PC—partner communication; MBEN—marital benefit measure; MCAT—perceived importance of medical care; PCAT—patient-centered care; PPAT—professional psychosocial services; MCEV—medical care evaluation; PCEV—patient-centered care evaluation. Scale scores are compared separately between two groups regarding pregnancy achievement (ANOVA).

**Table 3:** Parallel analysis results.

Factors	Real Data Component Eigenvalues	Simulated Data Mean Eigenvalues
Factor 1 *	7.884	2.597
Factor 2 *	5.633	2.415
Factor 3 *	4.068	2.292
Factor 4 *	2.851	2.163
Factor 5 *	2.625	2.065
Factor 6 *	2.175	1.959
Factor 7 *	2.027	1.896
Factor 8	1.731	1.827
Factor 9	1.572	1.741
Factor 10	1.438	1.663
Factor 11	1.209	1.584
Factor 12	1.123	1.526
Factor 13	1.075	1.467
Factor 14	1.016	1.401
Factor 15	0.976	1.338
Factor 16	0.924	1.282
Factor 17	0.808	1.227
Factor 18	0.769	1.164
Factor 19	0.741	1.119
Factor 20	0.666	1.075
Factor 21	0.637	1.032
Factor 22	0.586	0.976
Factor 23	0.539	0.929

* indicates which factors should be retained–in our case seven-factor structure.

**Table 4:** Regression model for association of COMPI scale domain scores and pregnancy achievement.

Scale Scores	Coefficient B	Coefficient Wald	*p*	Odds Ratio (OR)	Low 95% Confidence Interval for OR	High 95% Confidence Interval for OR
PD	−0.077	0.276	0.599	0.926	0.694	1.235
MD	0.628	7.927	0.005	1.874	1.210	2.901
SD	−0.125	0.529	0.467	0.882	0.630	1.236
AACOP	0.155	1.773	0.183	1.168	0.930	1.466
ACCOP	0.021	0.013	0.986	1.001	0.867	1.157
PACOP	−0.218	1.853	0.173	0.804	0.587	1.101
MBCOP	0.314	9.989	0.002	1.369	1.127	1.663
PC	1.791	3.481	0.042	2.442	1.927	4.277
ICS	0.145	1.111	0.292	1.156	0.883	1.512
MBEN	−0.277	2.414	0.120	0.758	0.535	1.075
MCAT	−1.816	0.690	0.406	0.163	0.002	1.808
PCAT	−0.864	0.339	0.561	0.421	0.023	7.738
PPAT	0.064	0.572	0.449	1.066	0.904	1.257
MCEV	0.808	0.300	0.584	2.243	0.125	4.253
PCEV	1.073	1.513	0.219	2.925	0.529	6.181
Beck Anxiety	0.084	2.273	0.132	1.087	0.975	1.213
Zung Depression	−0.025	0.212	0.645	0.975	0.875	1.086
Constant	−16.747	8.196	0.004	0.017		

Legend: PD—personal stress domain; MD—marital stress domain; SD—social stress domain; AACOP—active-avoidance coping; ACCOP—active-confronting strategies; PACOP—passive-avoidance strategies; MBCOP—meaning-based coping; ICS—infertility-related communication strategies; PC—partner communication; MBEN—marital benefit measure; MCAT—perceived importance of medical care; PCAT—patient-centered care; PPAT—professional psychosocial services; MCEV—medical care evaluation; PCEV—patient-centered care evaluation.

## Data Availability

Data are available upon request from the authors due to patient confidentiality regulations of our institution.

## Supplementary Materials

The following supporting information can be downloaded at https://www.mdpi.com/article/10.3390/healthcare14101429/s1, Table S1: Correlations of COMPI subscale and domain scores with BAI, ZDS and patient characteristics; Table S2: Correlations of COMPI scale domain scores.
