# Supplementary material for: Psychosocial Aspects of Infertility and Medically Assisted Reproduction in Serbia: A COMPI-Based Single Centre Study
Source: Healthcare (Basel). 2026 May 21;14(10):1429. doi: 10.3390/healthcare14101429 (PMC13207317; doi:10.3390/healthcare14101429)
Supplement: Supplementary file 1 [file healthcare-14-01429-s001.zip › healthcare-4272024-supplementary.pdf]

Supplementary Table S1. Correlations of COMPI subscale and domain scores with BAI, ZDS and patient characteristics.

Parameters	PD	MD	SD	AACOP	ACCOP	PACOP	MBCOP	PC	ICS	MBEN	MCAT	PCAT	PPAT	MCEV	PCEV
Pregnancy achievement	-0.069	0.245	-0.183	0.114	0.053	-0.015	0.300	0.202	0.122	0.010	0.049	0.032	0.138	0.054	0.104
	0.480	0.011	0.059	0.244	0.585	0.881	0.002	0.037	0.211	0.921	0.615	0.746	0.156	0.580	0.287
Beck Anxiety	0.152	-0.108	0.301	-0.151	0.106	0.036	0.235	-0.107	0.049	0.310	-0.125	-0.092	-0.073	-0.161	-0.059
	0.118	0.269	0.002	0.121	0.279	0.712	0.015	0.271	0.614	0.001	0.200	0.348	0.457	0.097	0.545
Zung Depression	-0.016	-0.091	0.359	-0.215	0.142	0.024	0.276	-0.257	-0.086	0.346	-0.115	-0.168	-0.153	-0.191	-0.079
	0.871	0.353	0.001	0.027	0.144	0.804	0.004	0.008	0.379	0.001	0.237	0.084	0.116	0.049	0.416
Age	0.128	0.058	-0.018	0.059	0.042	-0.021	-0.030	0.025	0.210	0.171	-0.095	0.019	0.095	-0.027	-0.064
	0.190	0.555	0.856	0.546	0.664	0.831	0.759	0.801	0.030	0.077	0.329	0.848	0.329	0.780	0.512
Education	0.019	-0.118	-0.015	-0.082	0.137	0.215	0.176	0.070	-0.037	0.123	-0.010	0.110	0.013	0.058	0.061
	0.846	0.225	0.880	0.399	0.159	0.026	0.070	0.471	0.708	0.206	0.922	0.261	0.890	0.550	0.533
Employment	0.029	-0.124	0.058	-0.103	0.152	0.066	0.011	-0.029	-0.014	0.036	-0.001	-0.032	-0.076	-0.011	0.069
	0.769	0.203	0.552	0.290	0.119	0.499	0.908	0.767	0.885	0.710	0.990	0.743	0.435	0.908	0.478
Relations	0.091	-0.211	0.067	0.157	0.011	0.130	-0.032	0.286	0.033	-0.093	0.083	0.140	0.156	0.138	0.137
	0.351	0.030	0.493	0.105	0.908	0.181	0.743	0.003	0.738	0.340	0.393	0.151	0.108	0.157	0.159
Pregnancy before	-0.067	0.217	-0.188	0.095	0.072	0.137	0.132	0.108	0.045	0.195	-0.030	0.018	-0.016	-0.002	-0.064
	0.491	0.025	0.053	0.333	0.463	0.159	0.175	0.270	0.644	0.044	0.760	0.856	0.867	0.984	0.514
Delivery before	0.055	0.074	-0.167	0.103	-0.191	0.099	0.025	0.138	-0.095	-0.014	-0.034	-0.043	-0.023	-0.046	-0.084
	0.575	0.450	0.085	0.289	0.049	0.313	0.798	0.155	0.328	0.889	0.730	0.663	0.816	0.636	0.392
Miscarriage before	-0.116	0.127	-0.050	0.158	0.163	0.035	0.199	0.041	0.113	0.281	-0.062	-0.003	-0.014	-0.014	-0.099
	0.236	0.193	0.606	0.103	0.093	0.720	0.039	0.674	0.246	0.003	0.523	0.976	0.884	0.885	0.309
Ectopic pregnancy before	-0.024	0.133	-0.076	-0.182	0.129	0.087	-0.075	-0.027	0.036	-0.028	0.077	0.090	0.018	0.074	0.128
	0.806	0.170	0.438	0.061	0.186	0.373	0.443	0.783	0.714	0.771	0.430	0.355	0.854	0.448	0.187
Previous pregnancy outcome	0.130	-0.141	-0.123	0.283	-0.344	-0.009	0.032	0.200	-0.183	-0.058	-0.098	-0.146	-0.022	-0.125	-0.198
	0.456	0.420	0.482	0.100	0.043	0.960	0.857	0.249	0.292	0.742	0.574	0.402	0.900	0.476	0.254
Infertility duration	-0.168	0.051	-0.100	-0.009	-0.076	0.042	0.001	-0.034	0.002	-0.103	0.002	0.057	0.159	0.033	-0.028
	0.084	0.601	0.307	0.925	0.437	0.664	0.991	0.725	0.980	0.291	0.980	0.556	0.101	0.738	0.775
Infertility cause	0.129	0.058	0.005	0.041	-0.117	-0.105	-0.116	0.074	0.074	0.069	0.005	0.118	0.140	0.080	0.040
	0.186	0.552	0.959	0.676	0.230	0.281	0.236	0.451	0.451	0.478	0.957	0.227	0.152	0.410	0.679

Infertility therapy	0.117	-0.078	0.102	-0.044	-0.412	-0.223	-0.323	0.009	0.135	-0.123	0.096	0.020	0.177	0.054	0.113
	0.229	0.426	0.294	0.656	0.001	0.021	0.001	0.928	0.167	0.206	0.323	0.840	0.068	0.581	0.247
Therapy type before	0.099	-0.005	0.019	-0.099	0.087	-0.002	0.140	0.079	-0.185	0.122	-0.004	0.134	-0.046	0.091	0.113
	0.388	0.968	0.872	0.391	0.449	0.984	0.221	0.490	0.105	0.288	0.969	0.241	0.690	0.429	0.326
Number of therapies	-0.014	0.064	0.123	0.113	-0.135	-0.122	-0.219	0.070	0.080	0.146	-0.065	-0.011	-0.088	-0.012	-0.060
	0.902	0.577	0.284	0.326	0.239	0.289	0.055	0.542	0.484	0.202	0.574	0.926	0.443	0.917	0.600
Pregnancy after therapy	-0.064	0.103	-0.317	0.163	-0.221	0.007	0.083	0.063	0.081	0.132	-0.123	-0.027	-0.041	-0.077	-0.145
	0.576	0.370	0.005	0.154	0.052	0.953	0.471	0.581	0.480	0.249	0.285	0.818	0.724	0.502	0.205
Therapy type now	-0.052	-0.202	-0.233	0.135	0.040	0.151	-0.162	0.100	-0.022	-0.111	0.091	0.226	0.061	0.197	0.134
	0.591	0.037	0.016	0.165	0.685	0.120	0.095	0.306	0.822	0.257	0.350	0.019	0.534	0.042	0.168

Supplementary Table S2. Correlations of COMPI scale domain scores

Domains	MD	SD	AACOP	ACCOP	PACOP	MBCOP	PC	ICS	MBEN	MCAT	PCAT	PPAT	MCEV	PCEV
PD	-0.070	0.255	-0.154	0.083	-0.088	-0.076	0.142	-0.090	0.098	-0.022	0.196	0.209	0.123	0.049
	0.476	0.008	0.113	0.397	0.368	0.439	0.145	0.355	0.316	0.820	0.043	0.031	0.208	0.616
MD		-0.247	0.055	-0.033	-0.104	-0.071	0.112	0.055	0.287	-0.028	-0.080	0.054	-0.047	-0.106
		0.010	0.574	0.737	0.288	0.467	0.250	0.572	0.003	0.771	0.413	0.584	0.631	0.275
SD			-0.240	0.006	-0.191	0.042	-0.273	-0.167	0.141	-0.088	0.036	-0.065	-0.040	0.019
			0.013	0.951	0.049	0.666	0.004	0.086	0.146	0.365	0.715	0.508	0.683	0.843
AACOP				0.059	0.242	-0.009	0.231	0.241	-0.065	0.002	-0.015	0.015	0.006	-0.032
				0.549	0.012	0.924	0.017	0.012	0.506	0.984	0.877	0.877	0.955	0.743
ACCOP					0.381	0.397	-0.108	-0.243	0.157	0.068	0.081	0.013	0.087	0.131
					0.001	0.001	0.269	0.012	0.107	0.488	0.405	0.897	0.374	0.178
PACOP						0.380	0.154	-0.140	0.052	0.066	-0.076	-0.074	-0.009	0.074
						0.001	0.114	0.150	0.594	0.500	0.439	0.447	0.931	0.450
MBCOP							0.028	-0.110	0.219	-0.064	-0.084	-0.060	-0.086	-0.013
							0.776	0.261	0.024	0.511	0.390	0.540	0.381	0.891
PC								0.120	-0.007	-0.040	-0.046	0.165	-0.015	-0.047
								0.218	0.940	0.679	0.636	0.090	0.875	0.632
ICS									0.119	-0.195	-0.194	0.030	-0.215	-0.218
									0.224	0.044	0.045	0.758	0.026	0.024
MBEN										-0.157	0.041	-0.014	-0.034	-0.151
										0.107	0.675	0.890	0.727	0.122
MCAT											0.510	0.391	0.815	0.933
											0.001	0.001	0.001	0.001
PCAT												0.560	0.902	0.633
												0.001	0.001	0.001
PPAT													0.559	0.407
													0.001	0.001
MCEV														0.855
														0.001
